# Development of the First Health-Related Quality of Life Questionnaires in Arabic for Women With Polycystic Ovary Syndrome (Part II): Dual-Center Validation of PCOSQoL-47 and PCOSQoL-42 Questionnaires

**DOI:** 10.7759/cureus.18060

**Published:** 2021-09-17

**Authors:** Samih A Odhaib, Fatemeh Nasiri Amiri, Mahmood Thamer Altemimi, Husam J Imran, Haider A Alidrisi, Miaad J Mohammed, Abbas A Mansour

**Affiliations:** 1 Adult Endocrinology, College of Medicine, University of Basrah, Basrah, IRQ; 2 Reproductive Health, Health Research Institute, Babol University of Medical Sciences, Babol, IRN; 3 Endocrinology, Thi Qar Specialized Diabetes, Endocrine and Metabolism Center, Thi-Qar, IRQ; 4 Diagnostic Radiology, Al-Refaee General Hospital, Thi-Qar, IRQ

**Keywords:** whoqol-bref, sexuality, quality of life, psychometrics, construct validity

## Abstract

Introduction

Validation assesses the acceptability, responsiveness, interpretability, and quality of any questionnaire in any specific population. This is done by correlation matrix evaluation of the proposed test tool with a previously well-validated assessment tool. The study objective is the dual-center assessment of the construct validity of the first health-related quality of life questionnaires for married and unmarried women with polycystic ovary syndrome (PCOS), i.e., PCOSQoL-47 and PCOSQoL-42, respectively.

Materials and methods

At two centers in Iraq, we enrolled 406 married women and 362 unmarried women with PCOS to test for the construct validity of PCOSQoL-47 and PCOSQoL-42, respectively, from August 2019-August 2020 (after obtaining full results of reliability testing in our previous work). We used the comparable domains from the multiculturally validated questionnaire (World Health Organization Quality of Life [WHOQOL-BREF]) as a comparator to assess the construct validity of the domains of the final highly reliable questionnaire drafts of PCOSQoL-47 and PCOSQoL-42 which were obtained from our previous work. The enrolled women will respond to WHOQOL-BREF and either PCOSQoL-47 or PCOSQoL-42, according to their marital status. Pearson's parametric correlational coefficient compared the total scores of the matched domains in one of our questionnaires and WHOQOL-BREF at p≤0.05. Values more than 0.3 denoted an important correlation between our test questionnaires and the well-validated WHOQOL-BREF questionnaire. The inter-rater reliability between our questionnaires and the comparator was calculated by Cronbach's alpha level, inter-item, and intra-class correlations coefficients matrix.

Results

We obtained a good respondent-to-item ratio of approximately 9:1 for both questionnaires. We had a good response for the domains of our questionnaires and WHOQOL-BREF. The coping domain at PCOSQoL-42 showed the highest Pearson's coefficient value of (0.708), which indicates a strong and significant correlation between the two constructs at (p<0.001). Other domains of the PCOSQoL-42 showed moderate significant correlation coefficient values. The psychological and emotional status domain of PCOSQoL-47 showed a weak yet significant correlation with its corresponding domain of the WHOHRQOL-BREF. The other domains of the PCOSQoL-47 showed moderate significant correlation coefficient values >0.5. The PCOSQoL-42 and PCOSQoL-47 showed high inter-rater reliability levels in measuring the requested construct or concept when we used Cronbach's alpha and inter-item correlation matrix assessment.

Conclusion

The individualized PCOSQoL-47 and PCOSQoL-42 for married and unmarried women with PCOS, respectively, represent the first reliable and valid HRQoL assessment tools for assessing the health-related quality of life (HRQoL) in those women with PCOS who use Arabic as a first or native language and address the sexual function as a separate domain.

## Introduction

To measure whether the inferences and conclusions of any questionnaire or test score are suitable for what they were designed to measure, we used the term "Validity" [1]. Validation assesses the acceptability, responsiveness, interpretability, and quality of any questionnaire in any specific population [2,3]. There is a crossover between reliability and validity testing during the psychometric assessment of a questionnaire [1].

The construct validation is essential in testing the quality of questionnaires that deal with outcomes that are not directly observable, like health-related quality of life (HRQoL). Lack of construct validity between different measures of the questionnaire renders the questionnaire's results difficult to interpret, with the inability to draw inferences or associations from its responses [4]. To test construct validity, the questionnaire of interest and a preexisting well-validated test score or questionnaire are administered to the same group of individuals. Correlation matrix analysis is used to assess the different forms of association between different measures which have the same construct (concept) in a convergent or a divergent manner [5].

In our previous work [6], we discussed the psychometric analysis of the two newly developed HRQoL questionnaires for married and unmarried women with polycystic ovary syndrome (PCOS), i.e., PCOSQoL-47 and PCOSQoL-42, respectively. We described the item pool formation, content and face validity, applicability in a pilot study, and then test-retest reliability evaluation along with internal consistency. In this article, we will discuss the validation analysis through the construct validity testing of the two new questionnaires through a real-life study in premenopausal women with PCOS in two endocrinology centers in Iraq.

## Materials and methods

This study had passed three phases to reach the final drafts of the questionnaire. The previous article [6] described the first two phases, in which all the psychometrics evaluation of the PCOSQoL-47 and PCOSQoL-42 questionnaires for married and unmarried women with PCOS, respectively.

Phase 3: The second recruitment (August 2019-August 2020)

After the conclusion of the test-retest reliability analysis and internal consistency, we used the third draft to measure the HRQoL in the women with PCOS from the two groups (Tables 1, 2), using the well-validated Arabic version of the WHOQOL-BREF [7] as a comparator for our questionnaires. A study group from two centers in Iraq performed the task of validity assessment for one year from August 2019-August 2020 (after obtaining full results of reliability testing in our previous work). These centers were Faiha Specialized Diabetes Endocrine and Metabolism Center (FDEMC), Basrah and Thi-Qar Specialized Diabetes Endocrine and Metabolism Center (TDEMC), Thi-Qar.

**Table 1:** The final draft for unmarried women with PCOS (PCOSQoL-42) after the end of test-retest reliability evaluation after correction of rank code. Adapted from Odhaib et al [6]. ^a^ Five-point Likert scale was already added to all items as follows: Always=1, Very Often=2, Quite Often=3, Seldom=4, and Never=5)

Domain^a^	Code	Items
Psychological and Emotional Status Domain	A1	Suffered from bad mood due to PCOS?
	A2	Felt pessimistic about the treatment?
	A3	Felt the urge to abandon treatments because of repetitive visits to doctors?
	A4	Felt frequent tantrums due to PCOS?
	A5	Experienced fear of diseases such as diabetes, hypertension and heart disease?
	A6	Experienced trouble dealing with others?
	A7	Suffered from low self-esteem due to PCOS?
	A8	Blamed yourself for having PCOS?
Menstrual Disorders and Fertility Domain	B1	Felt the need to decrease your weight to control PCOS?
	B2	Felt concerned about future infertility?
	B3	Felt concerned about the complete cessation of menstruation?
	B4	Felt concerned about menstruation at long intervals?
	B5	Felt the need to the regular need of oral contraceptive pills to control PCOS?
	B6	Felt you would accept all other PCOS manifestations if assured of pregnancy?
	B7	Experienced feelings of fear of cancer due to PCOS?
Body Image Domain	C1	Dissatisfied with some aspects of your appearance?
	C2	Tried to hide some flaws in your appearance?
	C3	Spend a significant amount of time checking your appearance in the mirror?
	C4	Ashamed of some part of your body?
	C5	Fear that others will discover flaws in your appearance?
	C6	Feel others are speaking negatively about your appearance?
	C7	Avoid looking at your appearance in the mirror?
Hair Disorders and Acne Domain	D1	Felt embarrassed about having excess facial and body hair?
	D2	Felt that alopecia is disturbing your appearance?
	D3	Felt concerned about the progression pattern of excess body and facial hair?
	D4	Felt that alopecia lead to decrease of your attraction and femininity?
	D5	Felt concerned about rapid re-growth of unwanted hair after its removal?
	D6	Always wear a headscarf to cover your hair due to alopecia?
	D7	Fear from facial acne to leave permanent scars?
	D8	Felt that acne is disturbing your appearance?
	D9	Felt that treatment of alopecia needs a long time and is worthless?
	D10	Felt the need to cover your body and face because of excess hair?
	D11	Avoid the social circumstances due to alopecia?
Coping Domain	E1	Try to consult a medical expert about what you think it is a flaw in your appearance?
	E2	Embarrassed to engage in social activities because of your appearance?
	E3	Compare your appearance with other women who you think they are more physically attractive than you?
	E4	Felt disappointed about the cure?
	E5	Avoidance of social circumstances due to excess body hair
	E6	Felt a lack of family support and acceptance of your disease?
	E7	Felt difficulty in communicating with other women who have PCOS?
	E8	Felt a lack of satisfaction with being a woman?
	E9	Concerned about your future role as a wife?

**Table 2:** The final draft for married women with PCOS (PCOSQoL-47) after the end of test-retest reliability evaluation after correction of rank code. Adapted from Odhaib et al [6]. ^a^ Five-point Likert scale was already added to all items as follows: Always=1, Very Often=2, Quite Often=3, Seldom=4, and Never=5) PCOS: polycystic ovary syndrome

Domain^a^	Item Code	Items
Psychological and Emotional Status	A1	Suffered from bad mood due to PCOS?
	A2	Felt easily tired?
	A3	Felt pessimistic about the treatment?
	A4	Felt the urge to abandon treatments because of repetitive visits to doctors?
	A5	Felt frequent tantrums due to PCOS?
	A6	Experienced trouble dealing with others?
	A7	Blamed yourself for having PCOS?
	A8	Experienced fear of diseases such as diabetes, hypertension and heart disease?
	A9	Suffered from low self-esteem due to PCOS?
Fertility and Sexual Life	B1	Felt fear of abortion?
	B2	Felt uselessness of sexual intercourse due to infertility?
	B3	Felt sad seeing pregnant women?
	B4	Experienced concern about future infertility?
	B5	Experienced fear of divorce or separation?
	B6	Felt sad seeing children?
	B7	Felt a lack of sexual desire?
	B8	Felt ashamed of sexual coldness/ unresponsiveness?
	B9	Felt unsatisfied with sexual life?
	B10	Experienced a lack of orgasm?
Body Image	C1	Try to consult a medical expert about what you think it is a flaw in your appearance?
	C2	Dissatisfied with some aspects of your appearance?
	C3	Tried to hide some flaws in your appearance?
	C4	Experienced fear of treatment complications?
	C5	Ashamed of some part of your body?
	C6	Spend a significant amount of time checking your appearance in the mirror?
	C7	Embarrassed to engage in social activities because of your appearance?
	C8	Feel others are speaking negatively about your appearance?
	C9	Compare your appearance with other women who you think they are more physically attractive than you?
	C10	Fear that others will discover flaws in your appearance?
	C11	Avoid looking at your appearance in the mirror?
Hair Disorders and Acne Domain	D1	Felt concerned about rapid re-growth of unwanted hair after its removal?
	D2	Felt concerned about the progression pattern of excess body and facial hair?
	D3	Felt that acne is disturbing your appearance?
	D4	Felt embarrassed about having excess facial and body hair?
	D5	Felt that alopecia lead to decrease of your attraction and femininity?
	D6	Felt that alopecia is disturbing your appearance?
	D7	Felt the need to cover your body and face because of excess hair?
	D8	Felt that treatment of alopecia needs a long time and is worthless?
	D9	Fear from facial acne to leave permanent scars?
	D10	Avoidance of social circumstances due to excess body hair?
	D11	Always wear a headscarf or a veil to cover your hair due to alopecia?
Obesity and Menstrual Disorders	E1	Felt concerned about a fast return to your previous weight after any weight loss?
	E2	Felt concerned about the complete cessation of menstruation?
	E3	Felt the need to the regular need of oral contraceptive pills to control PCOS?
	E4	Felt you would accept all other PCOS manifestations if assured of pregnancy?
	E5	Experienced feelings of fear of cancer due to PCOS?
	E6	Felt a lack of satisfaction with your current role as a wife?

The same previous enrollment criteria were applied during this recruitment phase, with the ethical consent forms signed [6]. Each woman received two questionnaires forums simultaneously, i.e., (PCOSQoL-47 or PCOSQoL-42, with the WHOQOL-BREF). All women received a full description of the questionnaire items by their interviewing endocrinologist.

WHOQOL-BREF

The 26-item WHOQOL-BREF is a cross-culturally validated, applicable generic questionnaire for assessing general healthy well-being [7]. It is not specific for assessing HRQoL in PCOS because of the heterogenic nature of the syndrome. The WHOQOL-BREF was used as a baseline or a comparator instrument to validate the questionnaires in different communities [8-11]. 

The WHOQOL-BREF has four domains of quality of life (QoL) - physical health, psychological, social relationships, and environmental - with the first two questions on overall QoL and general health. The questions reflected the respondent’s feelings in the last two weeks. Each question is scored on a five-point Likert scale, 1 indicates maximum HRQoL impairment, and 5 indicates the least impairment [9,12].

Construct validity

We used the construct validity testing to evaluate the ability of the present questionnaires to measure a construct (concepts) [13], by comparing the equivalent domains in the two questionnaires, i.e., PCOSQoL-47 or PCOSQoL-42, with the WHOQOL-BREF. The analysis was done using Pearson’s correlational analysis. A Likert scale measured the present questionnaire and the WHOQOL-BREF from 1 to 5. So, there was no need to create a specific syntax for the scores.

The first step in construct validity was to determine the corresponding similar domains that measure similar constructs and presumed statistically coherent outcomes from the two questionnaires, i.e., PCOSQoL-47 or PCOSQoL-42, and the WHOQOL-BREF (Table 3).

**Table 3:** The corresponding similar domains and items in the present questionnaires with WHOQOL-BREF. ^a^ The psychological domain questions are items (3, 4, 10, 15, 16, 17, and 18), the physical health domain questions are items (5, 6, 7, 11, 19, and 26), and the social relationships domain questions are items (20, 21, and 22). WHOQOL-BREF: World Health Organization Quality of Life instrument

Domains Codes	PCOSQoL-42 Domains	WHOQOL-BREF domains^a^	PCOSQoL-47 Domains	WHOQOL-BREF domains^a^
A	Emotional and psychological	Psychological	Emotional and psychological	Psychological
B	Menstrual irregularities and fertility	Social Relationship	Fertility and sexual life	Social Relationship
C	Body image	Physical health	Body image	Physical health
D	Hair and acne		Hair and acne	
E	Coping	Psychological	Obesity and menstrual irregularities	

Statistical analysis

For every respondent, there were scores of either PCOSQoL-47 or PCOSQoL-42 and the WHOQOL-BREF. The questionnaires' data were captured directly on an already prepared IBM SPSS Statistics for Windows, Version 26.0. (Armonk, NY: IBM Corp.) format by the interviewer. Then it was checked and compared by two other research members for consistency and accuracy of the data.

Pearson's correlational coefficient was used to compare the total score of the matched domains in our questionnaire and the WHOQOL-BREF and measured the significance level at a two-tailed significance of ≤0.05. The level of this coefficient determined the correlation between the two constructs where (0 - 0.19) very weak, (0.20 - 0.39) weak, (0.40 - 0.59) moderate, (0.60 - 0.79) strong, and (0.80 - 1.00) very strong. The inter-rater reliability between our questionnaires and the comparator was calculated by Cronbach's alpha level, inter-item, and intra-class correlations (ICC) coefficients matrix.

Management of Data

To achieve this goal, we enrolled all the women with PCOS from both groups who attended these two endocrine centers with a diagnosis of PCOS from August 2019 to August 2020, who fulfilled the enrollment criteria of the study, and consented for recruitment in the study [6].

There were 362 unmarried women with PCOS who responded to the PCOSQoL-42 and WHOQOL-BREF. There were 406 married women who responded to the PCOSQoL-47 and WHOQOL-BREF. All the responses were checked, registered, and calculated initially by two research members independently. The questionnaire paper and electronic forms for each woman from either group were numbered by the first author, where all other authors were blinded to, to achieve the responses' maximal anonymity.

The timeline, which was proposed initially by the authors to be a year period for construct validation analysis, determined the final sample size, the total number of 768 out of 913 women with PCOS (84.1%) who attended the two centers for management at a year period from August 2019 to August 2020. We excluded 145 women with PCOS from the study because they did not fulfill the enrollment criteria.

All the paper forums were sorted and stored according to registration numbers set by the first author, where they were ready to be retrieved on request from any respondent. All enrolled women were provided with a copy of their responses for their own to ensure transparency in dealing with their data. All women were told they would be free to withhold their consent at any time during the study till the time of final publication, and this would not affect by any way the level of medical care provided for them. No woman at all withheld her consent. There was no monetary incentive for the participants.

Ethical approval

All the study phases were in accordance with FDEMC ethical committee standards, from whom ethical approval was obtained for the study. The approval number was E43/3/2018. All enrolled women signed informed consent in Arabic before participating in the study.

## Results

The construct validation of the third draft

To start validation analysis, we combined our already measured PCOSQoL questionnaires with the corresponding similar domains in the Arabic version of the WHOQOL-BREF as a comparator, which was shown in Tables 1, 2, 3. We implemented a second recruitment study to include 362 unmarried and 406 married women with PCOS to respond to PCOSQoL-42 and PCOSQoL-47, respectively, along with the WHOQOL-BREF at the same time. The sample size had a respondent-to-item ratio of approximately 9:1.

The general characteristics of both cohorts and their initial responses are described in Tables 4, 5.

**Table 4:** General characteristics of the women with PCOS in the construct validation evaluation. BMI, body mass index; PCOS, polycystic ovary syndrome; SD, Standard Deviation; SE, Standard Error

Variables		PCOSQoL-42 (n=362)		PCOSQoL-47 (n=406)
Mean age (years) ± SD		22.59 ± 4.66		27.58 ± 6.09
Mean BMI (kg/m^2^) ± SD		28.63 ± 5.19		30.09 ± 6.14
Median duration of PCOS ± SE		3.00 ± .19		9.00 ± .31
Combined mean response time (minutes)	Mean ± SD	17.67 ± 4.00		20.04 ± 4.02
	Maximum	30		34
	Minimum	12		12
Assistance needed (%)		22 (6.10)		18 (4.4)

**Table 5:** Response evaluation of the women with PCOS in the construct validation evaluation to PCOSQoL-42, PCOSQoL-47, and the corresponding domains in WHOQOL-BREF. SD, Standard Deviation; PCOS, polycystic ovary syndrome; WHOQOL-BREF, World Health Organization Quality of Life instrument.

	Questionnaires	Domains	Respondents n (%)	Mean score ± SD	Median score	Minimum score	Maximum score
Unmarried women (n=362)	PCOSQoL-42	A (n=8)	333 (92.00)	19.36 ± 7.80	17	8	40
		B (n=7)	328 (90.61)	17.13 ± 6.68	15	7	35
		C (n=7)	351 (96.96)	16.83 ± 6.82	14	7	35
		D (n=11)	332 (91.71)	23.66 ± 8.03	22	11	52
		E (n=9)	325 (89.78)	23.59 ± 10.44	19	9	45
		Total (n=42)	281 (77.62)	98.36 ± 33.26	85	42	200
	WHOQOL-BREF	Psychological (n=7)	329 (90.88)	16.20 ± 4.99	15	7	32
		Physical Health (n=6)	335 (92.54)	14.24 ± 4.70	13	7	29
		Social Relationships (n=3)	288 (79.56)	6.61± 2.21	6	3	15
Married women (n=406)	PCOSQoL-47	A (n=9)	370 (91.13)	21.01 ± 8.20	19	9	45
		B (n=10)	282 (69.46)	25.36 ± 11.21	20	10	50
		C (n=11)	348 (85.71)	27.13 ± 10.75	22	11	54
		D (n=11)	366 (90.15)	24.19 ± 8.73	22	11	55
		E (n=6)	303 (74.63)	13.45 ± 4.48	12	6	30
		Total (n=47)	230(56.65)	104.89 ± 31.51	93	70	213
	WHOQOL-BREF	Psychological (n=7)	387 (95.32)	17.01 ± 5.01	16	10	32
		Physical Health (n=6)	389 (95.81)	15.97 ± 3.95	15	8	27
		Social Relationships (n=3)	387 (95.32)	7.70 ± 3.13	6	3	15

To perform the construct validation, we used the parametric analysis to estimate the Pearson's correlational coefficients at a two-tailed significance level of ≤0.05. Pearson's coefficient was used to compare the continuous variables like the total domain mean score. Values more than 0.3 denoted an important correlation between our test questionnaires and the well-validated WHOQOL-BREF questionnaire.

The coping domain showed the highest Pearson's coefficient value of (0.708), which indicates a strong correlation between the two constructs at the level of a two-tailed significance of <0.001. Other domains of the PCOSQoL-42 showed moderate significant correlation coefficient values >0.5. The psychological and emotional status domain of PCOSQoL-47 showed a weak yet significant correlation with its corresponding domain of the WHOHRQOL-BREF. The other domains of the PCOSQoL-47 showed moderate significant correlation coefficient values >0.5 (Table 6).

**Table 6:** Construct validity analysis of the PCOSQoL-42 and PCOSQoL-47 compared to the corresponding domains in the WHOQOL-BREF, using Pearson's correlational coefficient. WHOQOL-BREF, World Health Organization Quality of Life instrument

	Domains	Coefficient variables	WHOHRQOL-BREF domains		
			Psychological	Physical Health	Social Relationships
PCOSQoL-42	Psychological and Emotional Status (A)	Pearson Correlation	0.572 (moderate)		
		Sig. (2-tailed)	<0.001		
	Menstrual Disorders and Fertility (B)	Pearson Correlation			0.503 (moderate)
		Sig. (2-tailed)			<0.001
	Body Image (C)	Pearson Correlation		0.555 (moderate)	
		Sig. (2-tailed)		<0.001	
	Hair Disorders and Acne (D)	Pearson Correlation		0.527 (moderate)	
		Sig. (2-tailed)		<0.001	
	Coping (E)	Pearson Correlation	0.708 (strong)		
		Sig. (2-tailed)	<0.001		
PCOSQoL-47	Psychological and Emotional Status (A)	Pearson Correlation	0.301 (weak)		
		Sig. (2-tailed)	<0.001		
	Fertility and Sexual Life (B)	Pearson Correlation			0.556 (moderate)
		Sig. (2-tailed)			<0.001
	Body Image (C)	Pearson Correlation		0.594 (moderate)	
		Sig. (2-tailed)		<0.001	
	Hair Disorders and Acne (D)	Pearson Correlation		0.583 (moderate)	
		Sig. (2-tailed)		<0.001	
	Obesity and Menstrual Disorders (E)	Pearson Correlation		0.503 (moderate)	
		Sig. (2-tailed)		<0.001	

The inter-rater reliability analysis of the third draft

We used type A two-way mixed effect model inter-rater reliability and absolute agreement definition between our questionnaires and the comparator, through the use of Cronbach's alpha level and inter-item correlation (ICC) matrix with the same maneuver described in the test-retest reliability analysis in our previous paper [6]. The PCOSQoL-42 and PCOSQoL-47 showed high inter-rater reliability levels in measuring the requested construct or concept, as shown in Table 7.

**Table 7:** The internal consistency and reliability analysis of the third draft of PCOSQoL-42 and PCOSQoL-47. ^a^ The two-tailed significance level was less than 0.001. WHOQOL-BREF, World Health Organization Quality of Life instrument

Questionnaires	Domains	Respondents n (%)	Cronbach's Alpha	Inter-item correlation means	Intraclass correlation coefficient	95% confidence interval	
						Upper	Lower
PCOSQoL-42 vs. WHOHRQOL-BREF^a^	Psychological and Emotional Status (A) vs. Psychological	304 (84.0)	0.697	0.575	0.653	0.475	0.760
	Menstrual Disorders and Fertility (B) vs. Social Relationships	278 (76.8)	0.494	0.503	0.183	-0.141	0.456
	Body Image (C) vs. Physical Health	326 (90.1)	0.692	0.558	0.660	0.525	0.750
	Hair Disorders and Acne (D) vs. Physical Health	307 (84.8)	0.641	0.521	0.386	-0.191	0.676
	Coping (E) vs. Psychological	301 (83.1)	0.755	0.799	0.552	-0.165	0.796
	PCOSQoL-42 vs. WHOQoL-BREF	216 (59.7)	0.666	0.742	0.195	-0.107	0.511
PCOSQoL-47 vs. WHOHRQOL-BREF^a^	Psychological and Emotional Status (A) vs. Psychological	360 (88.7)	0.423	0.301	0.366	0.158	0.516
	Fertility and Sexual Life (B) vs. Social Relationships	265 (65.3)	0.456	0.556	0.168	-0.134-	0.427
	Body Image (C) vs. Physical Health	339 (83.5)	0.566	0.594	0.335	-0.164-	0.613
	Hair Disorders and Acne (D) vs. Physical Health	358 (88.2)	0.614	0.583	0.402	-0.150-	0.670
	Obesity and Menstrual Disorders (E) vs. Physical Health	295 (72.7)	0.665	0.503	0.606	0.376	0.736
	PCOSQoL-47 vs. WHOQoL-BREF	211 (52.0)	0.607	0.737	0.161	-0.103-	0.451

The inter-item correlations and ICC for the items each domain for all items in the domains in both questionnaires were >0.3, indicating good internal reliability of the dimensions, and implicated a highly significant relationship between the questionnaires' domains.

The final versions of PCOSQoL-42 and PCOSQoL-47 in Arabic are present as an appendix in our previous work [6].

## Discussion

Both five-domains PCOSQoL-42 and PCOSQoL-47 showed similar construct validity to that of the six-domain PCOSQ-50 when measuring the ICC values [14]. However, we used the WHOQOL-BREF as a comparator [7], while they used Short-Form 36 (SF-36) [13]. Both WHOQOL-BREF and SF-36 are reliable and internationally validated to measure the QoL in general [7,15]. This might change the corresponding domains and items between our scales with WHOQOL-BREF and that of PCOSQ-50 with the SF-36.

All domains in our scales showed significant correlations with the corresponding domains of WHOQOL-BREF. Four domains from each scale showed a moderately significant correlation; the coping domain of PCOSQoL-42 showed a strong or high correlation, and the psychological and emotional status domain of PCOSQoL-47 showed a weak correlation, yet highly significant at a two-tailed significance level <0.001 (Table 6).

The internal consistency of both questionnaires measured by the Cronbach's alpha in (Table 7) showed that only the coping domain of PCOSQoL-42 had exceeded the 0.7. Other domains had an alpha value ranging from (0.423 - 0.697), which could be considered a minor limitation. Yet, other parameters like the inter-item correlation mean and ICC were acceptable and highly significant. The WHOQOL-BREF had insufficient sensitivity in measuring the impact of PCOS symptoms because of the heterogeneity of the PCOS symptomatology [12]. Again this benefited our study that these domains showed a high validity for those domains that did not achieve the requested alpha value of 0.7. This would necessitate the comparison with other more sensitive scales in the subsequent multicenter nationwide studies to have better validity.

The sample size for the construct validity evaluation could be a strength and limitation at the same time. Although there were no absolute rules for the sample size needed to validate a questionnaire [16], we aimed for a sample size of 500 respondents for each questionnaire to meet the (very good sample size) proposed by Comrey et al. [17] to achieve an item to a respondent ratio more than 10:1. But we achieved only a ratio of 8.6:1, which was acceptable [16]. This was caused by the 35% drop-down of the number of attendees to the endocrine centers due to the SARS-CoV-2 pandemic, lockdown, and social distancing. The referral bias might be a limitation because the venue of the study represents two tertiary endocrine centers. We used a simple Arabic language with minimal use of ambiguous and medical terms to make the items more acceptable for women from both groups. We also avoided the words which might have different meanings and were confined to the original one-meaning words.

## Conclusions

The individualized PCOSQoL-47 and PCOSQoL-42 for married and married and unmarried women with PCOS represent the first reliable and valid HRQoL assessment tools for assessing the HRQoL in those women with PCOS who use Arabic as a first or native language,

Validation of PCOSQoL-47 and PCOSQoL-42 in other local languages like Kurdish will be undertaken following this study. Validation in local languages should be undertaken to cover a more diverse population.

The questionnaire’s longitudinal and discriminant validity also needs to be tested during treatment trials to test its sensitivity to the effect of interventions and change in our local population. This will enable us to use the questionnaire to measure the treatment effect on HRQoL and to compare the effects on HRQoL from different interventions.
